# The Treatment of Tubercular Enteritis

**Published:** 1893-09-02

**Authors:** G. A. Sutherland

**Affiliations:** Physician to the North London Consumption Hospital


### THE HOSPITAL CLINIC.

[*The Editor will be glad to receive offers of co-operation and contributions from members of the profession. All letters should be addressed to* THE EDITOR, THE LODGE, PORCHESTER SQUARE, LONDON, W.]

### THE TREATMENT OF TUBERCULAR ENTERITIS.

By G. A. SUTHERLAND, M.D.Ed., M.R.C.P.Lond., Physician to the North London Consumption Hospital.

Amongst the patients attending a consumption hospital a certain number present themselves who are suffering from diarrhœa due to tubercular ulceration of the intestine. This affection may be primary, there being no ascertainable disease in the other organs, or, as is more common, it may be secondary to tubercular lesions elsewhere, in the lungs, joints, &c. The patient is usually emaciated, and complains of diarrhœa, alternating with constipation, loss of appetite, flatulence, and abdominal pain. The motions are liquid or pultaceous, very offensive and frothy, and occasionally specks or streaks of blood are present in them. The following case illustrates the chief clinical symptoms in this disease, and also the good results which follow suitable treatment.

A girl, aged seven years, was brought to the hospital in July, 1892, suffering from weakness of the chest and diarrhœa. There was a strong family history of phthisis. The patient had had an attack of "consumption of the lungs" when three months old; had cut her teeth with bronchitis, and had always been subject to diarrhœa.

The present illness began three years previously, and was characterised by diarrhœa, loss of flesh, great weakness and lassitude, and a hacking cough. Her diet had been chiefly bread and butter and water, with occasionally mutton broth and Liebig's Extract of Meat. The appetite was very variable, at one time it would be ravenous, and she would keep on eating bread all day; at another it would be entirely lost. Sickness was frequently present, and the ingestion of food often brought on a loose action of the bowels, so that "everything passed right through her." Milk, eggs, and fruit were either rejected at once, or if retained, were not digested, and caused diarrhœa. The motions were fluid for the most part, occasionally lumpy, clayey, frothy, and offensive. Blood was present on a few occasions, and stringy masses (of mucus) were passed in large amount. The mother had already been at two hospitals with her child, and had been told she was suffering from ulceration of the lower bowel, and that meat and bread were to be excluded from the diet.

The patient was extremely emaciated, languid, and feeble. The complexion was waxy, with a marked flush on each cheek. The tongue was glazed, covered with a thin layer of epithelium, and of a bright red colour. There was slight œdema of the lower extremities. The abdomen was distended, resonant, and contained some small masses which were evidently fœcal. There was some impaired resonance at the apex of the right lung, with prolonged expiration, and scattered rhonchi were heard over both lungs, but no signs of active pulmonary mischief were present.

She was ordered a diet of lean meat, bread, and claret, and bismuth was prescribed in ten grain doses. In ten days there was perceptible improvement, she was brighter and more active, the diarrhœa was lessened, and the food was retained and digested. She had occasional pyrexial attacks, accompanied with sickness and cough, but these were of short duration, and yielded to rest in bed and careful dieting. The diarrhœa was very persistent, and any food outside the limit prescribed would at once induce a fresh attack. At the end of six months' treatment the child looked strong and robust. She had gained ten and a half pounds in weight, and was on ordinary diet, including porridge and milk. The bowels were moved as a rule once a day, and the motions were formed and contained no undigested materials.

I shall now proceed to consider the treatment under the following divisions: I. Dietetic; II. Medicinal; III. Local; IV. Hygienic.

I. *Dietetic.*—The previous food treatment employed has usually been what may be termed the ordinary diarrhœa diet, namely: Milk, milk puddings, and beef tea, and the result has been the steady progress of the disease, and the aggravation of the patient's symptoms. It must be recognised that in this affection there are present fermentative and putrefactive changes in the intestinal contents, and in consequence a catarrhal condition of the mucous membrane lining the bowel. Digestion and absorption are at a standstill, and the patient, although he may be swallowing large quantities of food, is being starved to death. The diet to be ordered must therefore satisfy the three following conditions: (1) It must be digestible; (2) it must be nourishing; and (3) it must be non-fermentable. All substances such as milk, fats, sugars, and starchy foods are to be excluded, as far as possible, in the first stage of treatment. I have obtained the best and most rapid results by putting the patient on a diet of lean meat. This may be given three times a day in the form of roast or boiled beef or mutton, chops and steaks. The meat should be as lightly cooked as the patient can tolerate it. Stale bread or toast is allowed along with the meat, but in as small a quantity as possible. In addition one or two glasses of sound claret are ordered with each meal. In many cases this diet is not only beneficial, but is actually enjoyed by the patient, who finds it a pleasant change from the slops he has been confined to for weeks or months. In other cases some latitude must be allowed, and fish, chicken, and the various proprietary meat essences, extracts, and jellies are permitted. In the case of children, pounded raw meat made up as a sandwich with thin slices of bread is very serviceable. The food, whether fluid or solid, is preferably administered cold, as anything hot is apt to induce increased peristalsis and diarrhœa. In the course of a week or ten days, guided by the condition of the motions, and the amount of catarrh still present, some additions to the albuminous diet may be allowed, such as milk, plain or peptonised, simple puddings, or cocoa and milk. These must be strictly regulated in amount to begin with, and any return of the diarrhœa, or the presence of undigested materials in the motions, necessitates a recurrence to the meat diet.

*(To be continued.)*

